# Circulating Monocytes Contribute to Erythrocyte Clearance in Polycythemia Vera

**DOI:** 10.3390/ijms26115133

**Published:** 2025-05-27

**Authors:** Marina D. Borges, Izabela F. Paes, Daniela P. Leonardo, Cristiane M. Souza, Dulcinéia M. Albuquerque, Carolina Lanaro, Katia B. B. Pagnano, Nicola Conran, Renata Sesti-Costa, Fernando F. Costa

**Affiliations:** Laboratory of Hemoglobin and Genome, Hematology and Hemotherapy Center, University of Campinas (UNICAMP), Campinas 13083-878, SP, Brazil; ma.borges@yahoo.com.br (M.D.B.);

**Keywords:** erythrophagocytosis, erythroblastic island, macrophages, myeloproliferative syndrome

## Abstract

Erythropoiesis is increased in polycythemia vera (PV), with proliferation of erythroid precursors, and macrophages from erythroblastic islands play a key role in this process. Circulating monocytes were shown to perform some of the macrophage’s functions in normal conditions, but their participation during stress erythropoiesis, as in PV, is yet to be determined. In this study, we evaluated the monocytes from the blood of healthy donors or PV patients regarding their phenotype, involvement in the clearance of erythroid cells, and their expression of iron-related molecules. We showed that circulating monocytes from PV patients contained red blood cell-derived material, which correlated with a reduction in Sirp-ɑ expression, indicating that they play a role in erythroid cell clearance in PV. Both PV monocytes and PV erythroid cells seem to influence the increase in erythrophagocytosis. The enhanced expression of heme-oxygenase-1 and ferroportin post-phagocytosis suggests their capability for heme degradation and externalization of residual iron. Moreover, PV monocytes presented higher expression of CD169, CD163, and VCAM-1, which are involved with erythroid adhesion, and they influenced in vitro erythroid cell line differentiation, suggesting that they may interfere with erythropoiesis in PV. Our findings highlight the similarities between PV monocytes and macrophages of erythroblastic islands. These insights contribute to a deeper understanding of erythrophagocytosis and erythropoiesis in the disease, offering new perspectives for advances in the field.

## 1. Introduction

Polycythemia vera (PV) is a myeloproliferative neoplasm triggered by mutations in the *JAK2* gene, which leads to a high production of erythroid cells, irrespective of erythropoietin (EPO). The result of this accelerated production is an increase in the population of morphologically normal blood cells through stress erythropoiesis (SE) [1,2,3,4,5,6]. The mutation also affects platelets and myeloid cells, including neutrophils and monocytes, suggesting that they may also undergo some changes. As opposed to SE in acute anemic states, which is a polyclonal and EPO-dependent physiological, transient, and adaptive compensatory response, chronic SE in PV is a maladaptive, clonal process driven by mutations in *JAK2* leading to autonomous, cytokine-independent erythroid proliferation, despite normal or low erythropoietin (EPO) levels [7,8,9].

Macrophages are an essential part of steady-state and stress erythropoiesis, as they promote the proliferation of erythroid cells, supply iron for their maturation, and capture and phagocytose the extruded nuclei during this process, which occurs in the erythroblastic islands (EBIs) [10,11,12]. These activities are, at least, partially dependent on the direct contact between macrophages and erythroid cells via the interaction of adhesion molecules [13]. PV patients have an increased population of bone marrow macrophages, correlated with high cell production [14]. The depletion of these cells in an animal model of PV reversed some of the main indicators of the disease, especially by normalizing the erythroid population, reducing splenomegaly, and decreasing extramedullary erythropoiesis, indicating their direct involvement in this scenario [15,16].

Recently, monocytes were shown to perform some of the functions of macrophages under normal conditions. These cells, especially the classical and intermediate monocyte subsets (C-MC and I-MC), seem to interfere with iron metabolism through the expression of ferroportin (FPN), ferritin, Sirp-α, and the receptor of transferrin [17]. They participate in the erythrophagocytosis, specifically C-MC, by clearing damaged and senescent RBCs from the circulation post-transfusion [18]. They directly promote erythroid proliferation, since the addition of monocytes in the erythroid culture promotes proliferation and diminishes apoptosis [19,20,21]. Whether monocytes play any part in erythrophagocytosis or erythropoiesis during chronic stress conditions is yet to be determined. Monocytosis in PV is known to be related to a higher risk of mortality, indicating alterations in these cells in the context of the disease [22].

This study aimed to investigate whether monocytes from patients with PV share some of the functions of EBI macrophages. Here, we report that these monocytes may adhere to erythroid cells, recycle iron, perform erythrophagocytosis in the circulation and in vitro, and overall, may influence erythropoiesis.

## 2. Results

### 2.1. Circulating Monocyte Subsets and Phagocytosis Markers Are Changed in PV Patients

**Figure 1:**
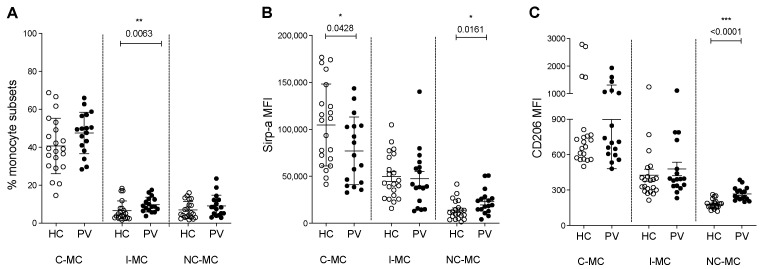
Characterization of circulating monocytes based on their subtype and expressions of Sirp-α and CD206. (**A**) Distribution of classical (C-MC), intermediate (I-MC), and non-classical (NC-MC) monocytes among PBMCs from healthy controls (HC) and polycythemia vera patients (PV). (**B**) Mean of fluorescence intensity (MFI) of Sirp-α in each monocyte subtype. (**C**) MFI of CD206 in each subtype. nHC = 21, nPV = 17. Mann–Whitney test, * < 0.05; ** < 0.01; *** < 0.001.

To investigate changes in the monocyte population in PV, we first analyzed the distribution of monocytes into the subsets classical (C-MC), intermediate (I-MC), and non-classical (NC-MC), and although no change was observed for C-MC and NC-MC, PV patients showed a higher percentage of I-MC in PBMCs when compared to HC (Figure 1A and Figure S1A). Investigating the expression of important molecules for the function of EBIs, we detected a reduction in Sirp-ɑ on C-MC from PV patients (Figure 1B and Figure S1B), suggesting a compromised ability to discern CD47 on healthy RBCs, potentially leading to their phagocytosis. We also observed an elevation of Sirp-ɑ, which not only plays a role in phagocytosis but also acts as a suppressor to the initiation of inflammatory signaling [23], in NC-MC from PV patients. This anti-inflammatory property was further suggested by the increase in M2 (regulatory monocyte) marker, CD206, on the NC-MC subset from PV patients when compared to HC (Figure 1C and Figure S1C).

### 2.2. Circulating Monocytes Play a Role in RBC Clearance in PV Patients

**Figure 2:**
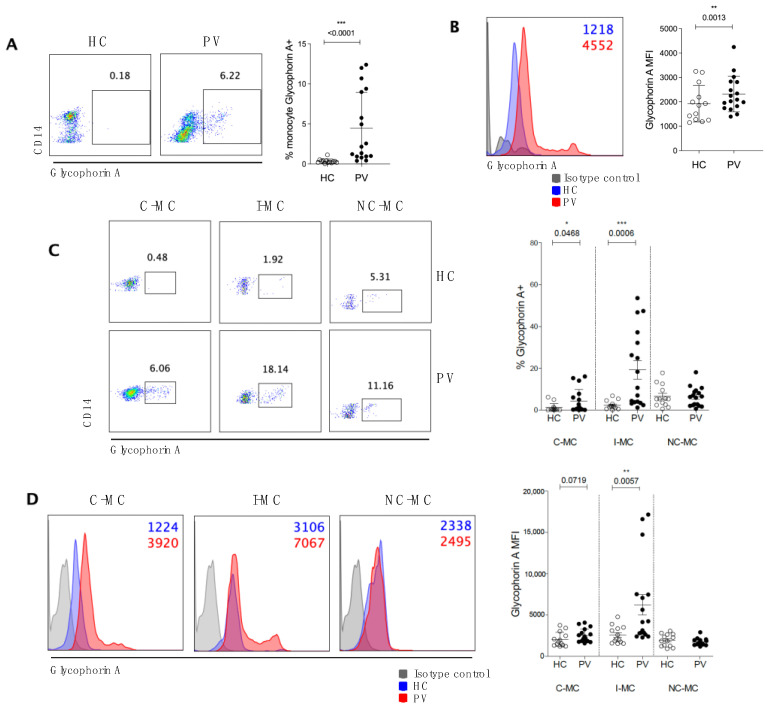
Phagocytosis of red blood cells by circulating monocytes of polycythemia vera patients (PV). Representative dot plots and percentage of total circulating monocytes (**A**) and subsets (**C**) of healthy controls (HC) and PV containing intracellular glycophorin A^+^. (**B**) representative histograms and mean fluorescence intensity (MFI) of glycophorin A inside total (**B**) and subsets (**D**) of monocytes. C-MC: classical monocytes; I-MC: intermediate monocytes; NC-MC: non-classical monocytes. nHC = 13, nPV = 17, Mann–Whitney test, * < 0.05; ** < 0.01; *** < 0.001.

Based on the reduced Sirp-α expression on monocytes from PV patients, we determined whether these circulating monocytes were involved in the removal of erythroid cells from the circulation. For this, we assessed the presence of intracellular glycophorin A in circulating monocytes. A mean of 0.35% of HC monocytes were positive for glycophorin A, whereas 4.8% of PV monocytes exhibited positivity for this erythroid marker (Figure 2A). The mean fluorescence intensity (MFI) of glycophorin A was also significantly higher in PV monocytes (Figure 2B), indicating that, in addition to a greater number of monocytes participating in phagocytosis, these cells also clear a considerably higher number of erythroid cells. The subtype of monocyte mainly found to have more RBC-derived material was I-MCs among all monocytes, and when comparing both groups of individuals, C-MC and I-MC from PV patients were able to uptake more RBCs than their counterparts from HC, as seen in the percentage and MFI of intracellular glycophorin A (Figure 2C,D). These findings suggest that these monocyte subsets play a role in erythrocyte clearance in PV.

### 2.3. Erythrophagocytosis Is Influenced by Both the Monocytes and RBCs of PV Patients

**Figure 3:**
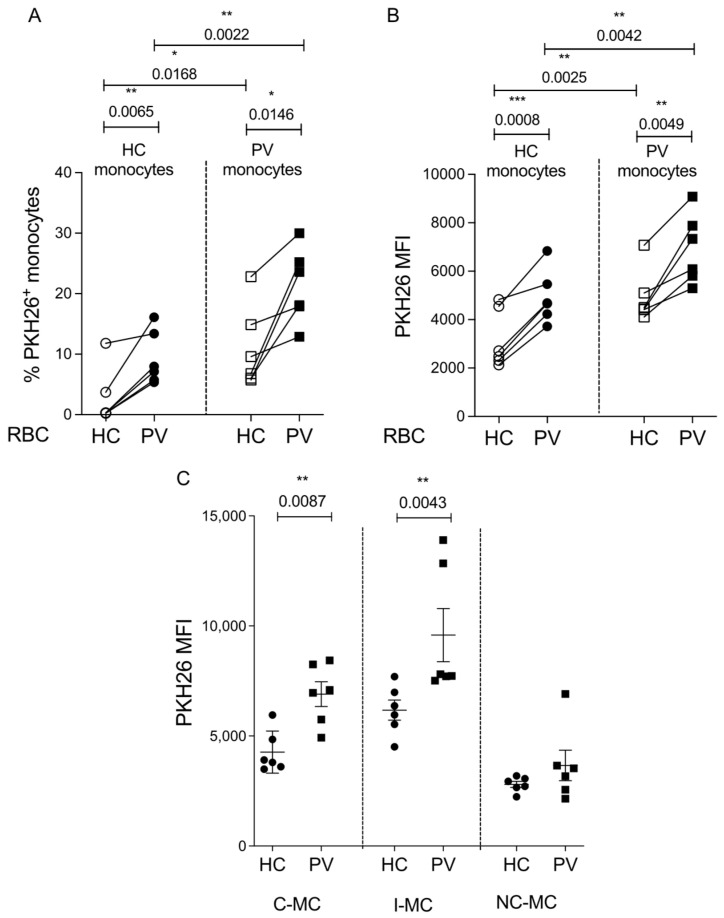
Monocytes and red blood cells from polycythemia vera patients (PV) participate in erythrophagocytosis in vitro. Red blood cells (RBCs) from healthy controls (HC) and PV previously stained with PKH26 dye were incubated with circulating monocytes from both groups for 2 h to allow phagocytosis. (**A**) Percentage of PKH26^+^ monocytes. Mean of fluorescence intensity (MFI) of PKH26 in total monocytes (**B**) and subsets (**C**). (**C**) Data from HC and PV monocytes incubated with PV RBCs. n = 6, Paired *t*-test, * < 0.05; ** < 0.01; *** < 0.001. Two-way ANOVA analysis with Tukey post-test shows *p* = 0.0051 for RBC factor and *p* = 0.0021 for monocyte factor on panel (**A**) and *p* = 0.0014 for RBC factor and *p* = 0.0019 for monocyte factor on panel (**B**).

To further confirm this possible role of PV monocytes in erythrophagocytosis, we performed an in vitro assay and observed the involvement of monocytes and RBCs in the phagocytic activity. For this, we co-incubated HC or PV monocytes with PKH26-stained HC or PV RBCs. PV monocytes exhibited an augmented phagocytic capacity for RBC from both HC and PV groups, and PV RBC-derived material was more readily taken up, regardless of monocyte origin, when compared to RBCs from HC individuals. This was evident in the percentage of PKH26^+^ (Figure 3A) and the MFI in the monocyte population (Figure 3B and Figure S2A). These findings indicate the mutual influence of monocytes and RBCs on the increased phagocytic and attachment capacity observed in PV patients. When monocyte subsets were discriminated, we can see that, in accord with the data from in vivo RBC uptake, C-MC and I-MC are the subsets with increased PKH26 MFI in monocytes from PV patients (Figure 3C and Figure S2B).

### 2.4. Expressions of HO-1 and FPN Are Increased Post-Erythrophagocytosis

**Figure 4:**
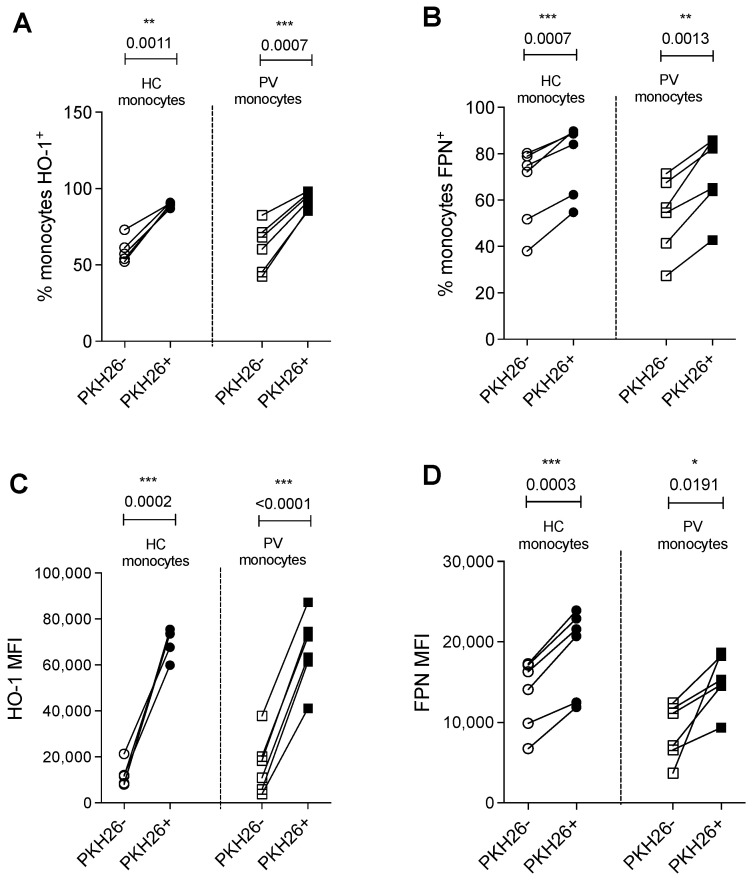
Expression of heme-oxigenase-1 (HO-1) and ferroportin (FPN) after phagocytosis of red blood cells. Percentage of HO-1^+^ (**A**) and FPN^+^ (**B**) monocytes from healthy controls (HC) and PV on PKH26^−^ and PKH26^+^ groups; mean of fluorescence intensity (MFI) of HO-1 (**C**) and FPN (**D**) on HC and PV monocytes on PKH26^−^ and PKH26^+^ groups. n = 6, paired *t* test, * < 0.05; ** < 0.01; *** < 0.001.

After the observation of enhanced phagocytosis by PV monocytes, we proceeded to investigate the implications on iron metabolism. By comparing monocytes that participated in the phagocytosis of PV RBC (PKH26^+^) to those not involved in the process (PKH26^−^), we noted a consistent increase in both the percentage of monocytes expressing HO-1 and ferroportin (FPN) (Figure 4A,B) and the amount of HO-1 and FPN per monocyte PKH26^+^, as seen by MFI (Figure 4C,D and Figure S3A,B), regardless of the origin of monocytes and RBCs. These findings suggest the capability of monocytes for heme degradation and the externalization of residual iron after engulfment of erythrocytes. Furthermore, we observed a trend toward a reduction in FPN expression in PV monocytes compared to HC monocytes prior to phagocytosis, suggesting that these monocytes may be maintaining iron in intracellular storage rather than externalizing it.

### 2.5. Monocytes from PV Patients Express Adhesion Molecules for Erythroid Cells and May Interfere with Erythropoiesis

**Figure 5:**
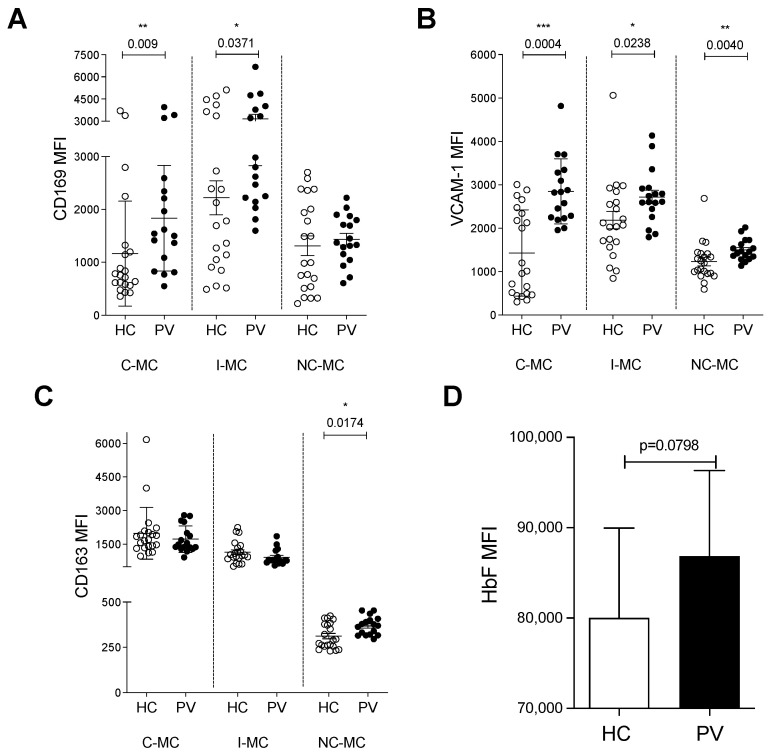
Monocytes from polycythemia vera patients (PV) may interact with erythroid cells. Expression of CD169 (**A**), VCAM-1 (**B**), and CD163 (**C**) on circulating monocytes of healthy controls (HC) and PV (nHC = 21, nPV = 17, Mann–Whitney test, *p* < 0.05). (**D**) Mean of fluorescence intensity (MFI) of fetal hemoglobin (HbF) in K562 cells co-cultured with HC or PV monocytes—data are representative of 3 independent experiments (n = 6, paired *t* test, * < 0.05; ** < 0.01; *** < 0.001).

Considering the involvement of PV monocytes with erythrophagocytosis and iron metabolism, we set out to find whether they were able to interact with erythroid cells through adhesion molecules expressed by EBI macrophages. CD169 exhibited higher expression levels in C-MC and I-MC subsets of PV patients (Figure 5A and Figure S4A), whereas VCAM-1 was more prevalent in all subsets compared to HC cells (Figure 5B and Figure S4B). CD163, which acts both as an adhesion molecule and as the receptor of the hemoglobin–haptoglobin complex, participating in iron metabolism as well as in the formation of EBIs, was elevated in NC-MC of PV patients (Figure 5C and Figure S4C). These findings suggest that circulating monocytes from PV patients possess the necessary molecular components for adherence to erythroid cells, similarly to EBI macrophages.

To evaluate whether monocytes are able to influence erythroid differentiation, we cultured K562 cells, a well-established cell line that can undergo erythroid differentiation in response to hemin [24], in the presence of monocytes from HC or PV patients. Differentiation was measured by the intracellular HbF concentration. We observed a trend of PV monocytes to induce a higher production of HbF in K562 cells when compared to HC monocytes (Figure 5D and Figure S4D), although not statistically significant. Taken together, the data suggest that PV monocytes may interfere with erythropoiesis in a way that HC monocytes do not. However, more experiments must be performed to clarify whether the monocytes influence RBC differentiation, especially when using co-culture with CD34-derived erythroid cells.

## 3. Discussion

By investigating circulating monocytes from patients diagnosed with PV, we noted an increased presence of I-MC, in accordance with previously published studies, in which PV patients had increased populations of I-MC and NC-MC, which are considered to be the inducers of the inflammatory response [7,25]. Additionally, we show, for the first time, the uptake of RBC material by circulating monocytes from patients with PV, a function primarily attributed to macrophages.

Our data suggest that both monocytes and erythrocytes influence erythrophagocytosis in PV. Studies by Wang and colleagues [26] demonstrated that erythrocytes with the *JAK2* V617F mutation exhibit reduced CD47 expression, leading to a greater susceptibility for phagocytosis by macrophages, with or without *JAK2* mutations, compared to healthy erythrocytes. Also, RBCs might be affected by their constitutive overproduction in these patients, which may cause the rapid production and compromised differentiation of erythrocytes, with an increase in apoptotic cells. These events highlight the involvement of PV RBCs in the erythrophagocytosis process. In addition, our data show a reduction in Sirp-α expression by C-MC in PV patients, which may account for the involvement of this subset of monocytes in erythrophagocytosis in the disease. However, additional mechanisms other than Sirp-α may account for superior RBC ingestion, especially in I-MC. VCAM-1, which is raised in all monocyte subsets in PV, or other adhesion molecules, may also play a part in this process. The changes in Sirp-ɑ, VCAM-1, and other adhesion molecules’ expression by monocytes may be affected by the *JAK2* mutation [7], causing shifts in their activity. In 2023, Lysenko and colleagues [27] demonstrated that the interruption of the interaction between CD47 and Sirp-α in an animal model of PV ameliorates the disease phenotype and that the anti-CD47 treatment resulted in the expansion of monocyte-derived macrophages and dendritic cells with phagocytic phenotype. *JAK2* mutant macrophages display a “pro-phagocytic” function that contributes to the correction of the disease in the absence of CD47-Sirp-α interactions. Future investigations could determine if the presence of the *JAK2* mutation across the myeloid lineage, including monocytes, is associated with altered cellular functions.

The increased presence of HO-1 and FPN in monocytes after RBC uptake suggests internalization of RBC material and indicates enhanced capabilities to catabolize heme released from engulfed erythrocytes and export the resulting iron. The upregulation of iron metabolism molecules post-phagocytosis suggests a significant influence of monocytes on iron availability to meet the demand for erythroid cell production. The trend toward a positive influence of monocytes from PV patients on the erythroid differentiation of K562 cells reinforces this hypothesis. It has previously been shown that the presence of PV macrophages in primary CD34^+^ erythroid cell cultures increases differentiation [15]. Monocytes are known to influence the differentiation of CD34^+^ cells to erythroid cells [21]. This observed influence of PV monocytes, particularly in K562 cells, a lineage that naturally differentiates in the presence of hemin [24,28], although small, suggests their potential involvement in the erythroid differentiation process, contributing, together with macrophages, to the greater demand for erythrophagocytosis and erythropoiesis in PV compared to homeostatic situations. K562 cell line is widely established as a model of erythroid progenitors used in many studies [29,30,31,32,33,34,35], having been used to investigate the influence of macrophages in erythroid maturation [36]. Even though it is commonly used, and the results can be extrapolated to primary erythroid cells, experiments in patients’ primary cells should be carried out to further support this influence and clarify the role of PV monocytes in the proliferation and differentiation of erythroid cells.

Macrophages play a crucial role during erythropoiesis via the formation of EBIs, directly influencing the proliferation of erythroid cells [16,37,38,39,40,41]. Their significance becomes even more pronounced in situations of SE, such as that observed in PV [7,42]. In the current study, we investigated circulating monocytes in these patients, and an upregulation of molecules related to the phenotype of the EBI macrophages was noted, particularly adhesion molecules for erythroid cells and those of an anti-inflammatory profile, with NC-MC showing the highest similarity. This suggests their potential influence on processes within this niche, indicating distinct roles for monocyte subsets in the process.

Many studies show transcriptomic differences between PV and healthy individual cells. Van Egeran et al. [43] demonstrated the pathogenic proinflammatory, profibrotic phenotype in the bone marrow monocyte, which are mostly of the intermediate phenotype, while Fan et al. [44] found significant transcriptional differences of monocytes/macrophages in the bone marrow of PV patients when compared to healthy control bone marrow with an increase in CD14^+^CD16^+^ and CD163^+^ cells. These data highlight the unique profile of PV monocytes.

Depleting EBI macrophages in a PV animal model led to improvements in various disease aspects, including splenomegaly, reticulocytosis, erythrocytosis, and elevated hematocrit [15,16]. Monocytes are known to be recruited to the spleen during stress situations for differentiation into macrophages [45]. Therefore, depleting monocytes in PV animal models could reveal the extent of their involvement in disease processes and their therapeutic potential.

One of the major limitations of this study was the prevalent use of HU by most patients, preventing a comparison between treated and untreated groups. This comparison is crucial due to the documented influence of medications on cell phenotypes, the subset distribution of monocytes, and their direct impact on inflammation and hemolysis [46]. HU is also known for modulating the expression of several adhesion molecules on RBCs, but evidence shows that it contributes by reducing the expression of molecules such as α4β1, CD36, VLA-4, and ICAM-4 of erythroid cells from sickle cell disease patients. The increase in CD47 during HU therapy shows the importance of considering the influence of HU on the interaction between erythroid cells and monocytes [47,48,49]. Despite this limitation, treatment did not align the expression profile in the cells of patients with that of healthy individuals.

We acknowledge the challenges inherent in an observational approach, particularly its inability to establish definitive causal relationships. Observational studies remain a cornerstone in advancing medical knowledge, especially when investigating complex diseases like PV. We also acknowledge that the in vitro phagocytosis assay may not reflect the same exact conditions as the in vivo situation, and it has its limitations. Despite that, it is still a good way to test the cell behavior in an isolated system. Our findings provide a new perspective on the role of monocytes in PV and provide data that could guide future experimental work. Future experiments will build on this foundation to elucidate mechanisms by addressing key questions raised by our findings, such as the influence of PV monocytes on primary erythroid cells; the influence of HU treatment; whether monocytes adhere to erythroid cells in different stages of development, phagocyte them, or achieve both; the molecular mechanisms leading to erythrophagocytosis; and many others.

Taken together, our findings suggest that monocytes in patients with PV exhibit greater similarity to EBI macrophages than monocytes in healthy conditions, indicating an increased capacity to influence processes within this niche. Our data also expand the understanding of how the excessive RBCs in PV can be cleared beyond the macrophage. The identification of circulating monocytes as contributors to erythrophagocytosis in the disease and Sirp-α-CD47 interaction as a possible mechanism involved in the process underscores a previously underappreciated mechanism of RBC turnover, which may play a role in disease progression, offering new avenues for therapeutic targeting.

## 4. Materials and Methods

### 4.1. Sample Collection

PV patients treated at the Blood Center of the University of Campinas, UNICAMP, were recruited in different stages of their treatment, all in a stable and controlled clinical state. 91% of patients received hydroxyurea (HU) treatment. Healthy controls (HCs) were blood donors from the same institution, recruited after being approved for donation and matched by age and sex with the patients. The study was conducted in accordance with the Declaration of Helsinki and approved by UNICAMP Human Research Ethics Committee (protocol number CAAE: 88768318.6.0000.5404, date of approval: 7 February 2018). All participants agreed to participate and signed informed consent forms. Demographics are presented in Table 1.

### 4.2. Peripheral Blood Mononuclear Cells’ Isolation

Whole blood (20 mL) was collected from each participant in lithium heparin tubes for the isolation of mononuclear cells. Under a laminar flow, the blood was diluted in sterile phosphate-buffered saline (PBS) to a final volume of 50 mL. The diluted blood was carefully layered over Ficoll-Paque Plus (density 1077 g/mL—Cytiva, Uppsala, Sweden) in a 2:1 ratio, avoiding mixing of the phases to allow density-based separation via centrifugation at 400× *g* for 30 min at room temperature, with no brake or acceleration. The peripheral blood mononuclear cell (PBMC) layer was collected using a Pasteur pipette and transferred to a new tube for two washing steps with PBS at 1500 rpm for 10 min. The pellet obtained was resuspended in 5 mL of chilled RBC lysis buffer (Roche Diagnostics, Mannheim, Germany) and incubated for 10 min at 4 °C to lyse erythrocytes. Following this, the volume was completed with PBS and centrifuged again. The resulting pellet was resuspended in 1mL of PBS for mononuclear cell counting using a Neubauer chamber.

### 4.3. Monocyte Phenotyping via Flow Cytometry (FC)

The previously isolated PBMCs were utilized for the phenotypic analysis of circulating monocytes by FC using Cytoflex equipment (Beckman Coulter, Brea, CA, USA). Based on the expression of CD14 and CD16, they were gated and separated into the following subsets: classical (C-MC and CD14^++^CD16^−^), intermediate (I-MC and CD14^++^CD16^+^), and non-classical (NC-MC and CD14^+^CD16^+^) (Figure S1). The expression of targeted molecules (Sirp-α, CD206, VCAM-1, CD163, and CD169) was evaluated using fluorescent antibodies that were incubated with cells for 30 min at 4 °C, followed by a wash and resuspension in PBS-BSA-ACD. The in vivo phagocytosis analysis was based on the experiment described by Li et al. [18]. Briefly, after monocytes were stained with anti-CD14 and CD16 antibodies, cells were fixed and permeabilized with Cytofix/Cytoperm kit (BD Biosciences, Franklin Lakes, NJ, USA), according to the manufacture’s instruction, for the following staining with intracellular anti-glycophorin A antibody for 30 min at 4 °C, followed by wash with PBS. All data were analyzed using FlowJo software version 10.9.0 (BD Biosciences, Franklin Lakes, NJ, USA). Antibodies’ information is described in Table S1.

### 4.4. In Vitro Phagocytosis Assay

The methods of Haschka et al. [17] were adapted as follows. Monocytes were isolated from PBMCs using anti-CD14 magnetic beads (Miltenyi Biotec, Bergisch Gladbach, Germany), according to the manufacturer’s instructions. Briefly, PBMCs were resuspended in PBS-BSA-ACD and were incubated with magnetic beads for 15 min at 4 °C. After incubation, PBS-BSA-ACD was added for washing step via centrifugation at 1500 rpm for 10 min. The pellet was resuspended in 1 mL of PBS-BSA-ACD and applied to LS columns (MACS) placed on a magnetic stand and allowed to flow through. After three washes of 3 mL of PBS-BSA-ACD, the column containing the CD14^+^ cells was removed from the magnetic stand, and the cells were eluted in 6 mL of PBS-BSA-ACD and removed from the column using a plunger. Following centrifugation, the final pellet was resuspended in RPMI-1640 culture medium (Sigma-Aldrich, St. Louis, MO, USA) for cell counting in a Neubauer chamber.

Cells were incubated overnight at 37 °C with 5% CO_2_ in a 24-well plate containing 500 μL of RPMI-1640 medium, at a concentration of 5 × 10⁵ cells per well, and allowed to adhere to the bottom of the plate. The following day, RBCs were isolated from peripheral blood collected in EDTA tubes. Two washes were performed using PBS for 2 min at 1600 rpm using 80 μL of blood. From the resulting pellet, 20 μL was separated for cell count and use. Following the manufacturer’s instructions, RBCs were stained with the membrane dye, PHK26 (Sigma-Aldrich). Briefly, the cells and the dye were resuspended in Diluent C and added to the same 15 mL polypropylene conical tubes for four minutes at room temperature, being sporadically mixed. Inactivated fetal bovine serum (FBS) was added to stop the staining, followed by centrifugation at 400× *g* for 10 min. Cells were washed 3 times and resuspended in complete medium (RPMI-1640 (Corning, Corning, NY, USA), 10% FBS, 1% L-glutamine, and 1% penicillin/streptomycin). Stained RBCs were then added to each well at a density of 5 × 10^6^ cells (1:10), at final volume of 1ml of complete medium, and allowed to interact with monocytes for two hours. After this period, cells were collected using chilled PBS and washed at 1500 rpm for 10 min. Chilled lysis buffer was added for 10 min at 4 °C to eliminate any non-phagocytosed RBCs. Following wash with PBS, the resulting pellets were resuspended in PBS-BSA-ACD with the addition of Fc block (BD Biosciences) and incubation for 10 min at room temperature. Cells were divided into two staining panels for FC.

For first panel, the primary anti-FPN antibody was added, followed by a wash at 7000 rpm for two minutes. The secondary antibody was then added and incubated. After another wash, anti-CD14 and anti-CD16 antibodies were incubated, followed by another wash. The final pellet was resuspended in 300 μL for acquisition via flow cytometry. All incubations were performed for 30 min at 4 °C. For the second panel, anti-CD14 and anti-CD16 antibodies were first added. After washing, the Cytofix/Cytoperm kit was used for cell fixation and permeabilization, according to the manufacturer. Finally, cells were incubated with anti-HO-1 for 30 min at 4 °C, followed by a wash and analysis by conventional flow cytometry.

### 4.5. K562 Culture

The K562 cell line (ATCC—CCL-243) was co-cultured with CD14^+^ cells from both HC and PV individuals for 48 h in the presence of 30 µM of hemin (Sigma-Aldrich) to induce erythroid differentiation. Cells were maintained for 48 h in a 24-well plate containing 1.1 mL of complete medium (DMEM (Gibco, Waltham, MA, USA), 10% FBS, and 1% penicillin/streptomycin) at 37 °C with 5% CO_2_ at a density of 10^5^ K562 and 10^4^ CD14^+^. After, K562 cells were harvested and stained for intracellular fetal hemoglobin (HbF) using an anti-HbF antibody, followed by acquisition by FC.

### 4.6. Statistical Analyses

Prism 5.1 software (GraphPad) was used for statistical analysis, and *p* values < 0.05 were considered statistically significant. The results are expressed as mean ± standard deviation. Statistical significance was calculated using two-tailed paired *t*-test or Mann–Whitney test, as indicated in the figure legends. Two-way ANOVA with repeated/paired measures and Tukey post-test was performed for comparisons with more than two groups.

## 5. Conclusions

Our findings highlight the similarities between circulating monocytes in patients with PV and the macrophages within EBIs, as evidenced by their shared expression of molecules capable of interacting with erythroid cells. Even though macrophages play the primary function in phagocytosing damaged or senescent RBCs, circulating monocytes play a role in this process under stress conditions. This activity seems to be influenced by the characteristics of both erythrocytes and monocytes. The observed features of monocytes in stress erythropoiesis suggest their potential involvement in processes occurring within EBIs, such as erythropoiesis, iron supply, and erythrophagocytosis. These insights contribute to a deeper understanding of the complex interplay between monocytes and erythroid cells in the disease, offering new perspectives for future research and potential therapeutic interventions targeting this intricate cellular crosstalk.

## Figures and Tables

**Table 1:** Summary of the main demographic, hematological, and clinical characteristics of polycythemia vera patient population.

	**Median (Min–Max)**
Age (n = 37)	63 (24–77)
RBC (3.96 × 10^6^/μL) (n = 37)	4 (2.7–8.1)
Platelets (130–400 × 10^3^/μL) (n = 37)	318 (137–898)
WBC (3.7–11.1 × 10^3^/μL) (n = 37)	5.47 (2.4–16.3)
MHC (27.3–32.6 pg) (n = 37)	33.8 (17.8–43.3)
MCV (82–98 fL) (n = 37)	104.6 (65.4–124.6)
HGB (11.8–16.7 g/dL) (n = 37)	14 (10.2–17.7)
HCT (36–50%) (n = 37)	42.75 (33.8–55)
Monocytes (0.2–0.92 × 10^3^/μL) (n = 37)	0.25 (0.09–0.7)
Serum iron (70–180 μg/dL) (n = 18)	75.5 (23–143)
Ferritin (m: 30–400; f: 13–150 ng/dL) (n = 18)	58.5 (12.4–429.8)
TIBC (255–450 μg/dL) (n = 18)	359.5 (261–630)
Transferrin saturation (20–45%) (n = 18)	20 (4–37)
	**n**
Sex (male/female) (n = 37)	19/18
Hydroxyrea (n = 37)	34
Phlebotomy (n = 37)	3
Presence of JAK2 V617F mutation (n = 37)	35
Presence of JAK2 exon12 mutation (n = 37)	2

RBCs: red blood cells; WBCs: white blood cells; MCH: mean corpuscular hemoglobin; MCV: mean corpuscular volume; HGB: hemoglobin; HCT: hematocrit; TIBC: total iron-binding capacity.

## Data Availability

The data presented in this study are available upon request from the corresponding author.

## Supplementary Materials

The following supporting information can be downloaded at: https://www.mdpi.com/article/10.3390/ijms26115133/s1.
