# Supplementary material for: Circulating Monocytes Contribute to Erythrocyte Clearance in Polycythemia Vera
Source: Int J Mol Sci. 2025 May 27;26(11):5133. doi: 10.3390/ijms26115133 (PMC12155271; doi:10.3390/ijms26115133)
Supplement: Supplementary file 1 [file ijms-26-05133-s001.zip › ijms-3489899-supplementary.pdf]

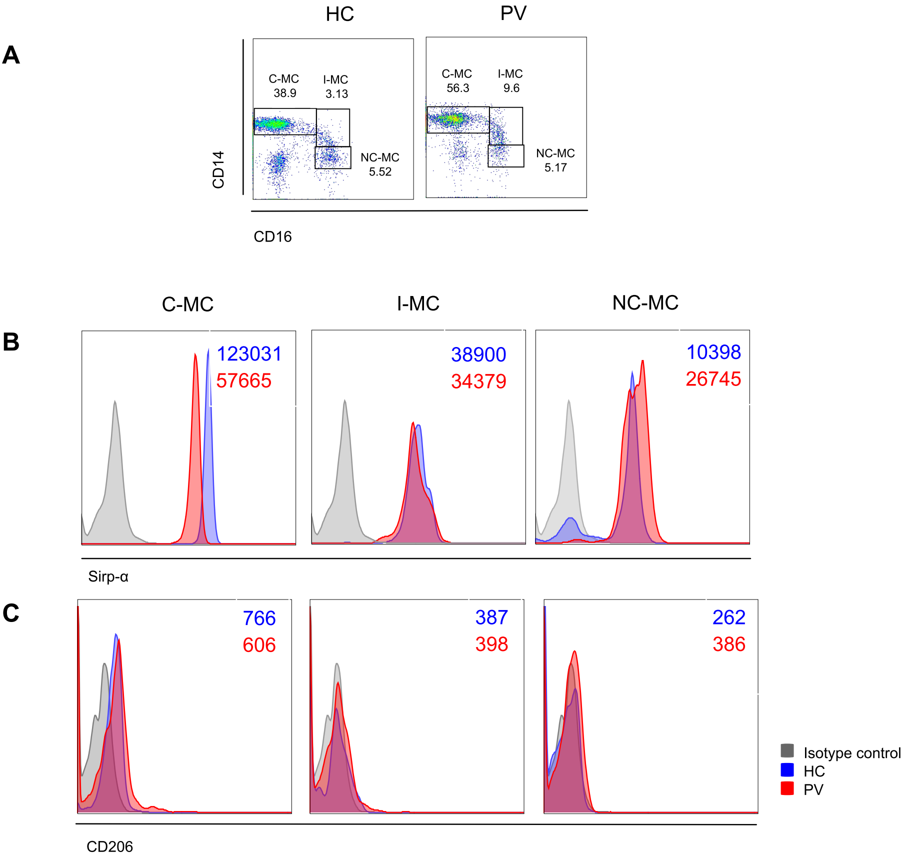
A
HC
PV
C-MC
38.9
I-MC
3.13
NC-MC
5.52
C-MC
56.3
I-MC
9.6
NC-MC
5.17
CD14
CD16
C-MC
I-MC
NC-MC
B
123031
57665
38900
34379
10398
26745
Sirp-α
C
766
606
387
398
262
386
CD206
Isotype control
HC
PV

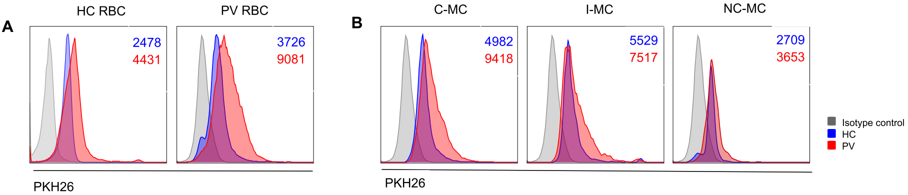
A
HC RBC
2478
4431
PV RBC
3726
9081
PKH26
B
C-MC
4982
9418
I-MC
5529
7517
NC-MC
2709
3653
PKH26
Isotype control
HC
PV

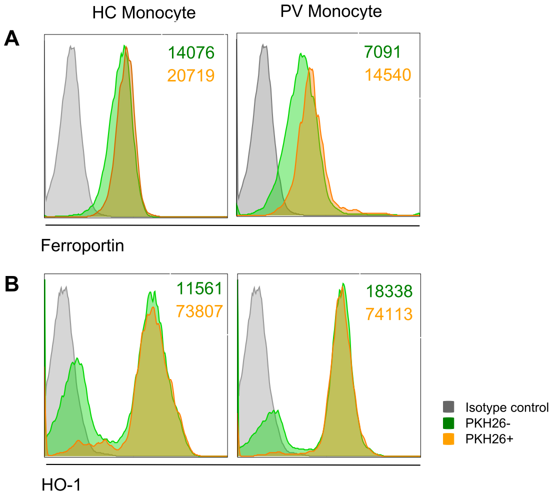

HC Monocyte
PV Monocyte
A
14076
20719
7091
14540
Ferroportin
B
11561
73807
18338
74113
HO-1
Isotype control
PKH26-
PKH26+

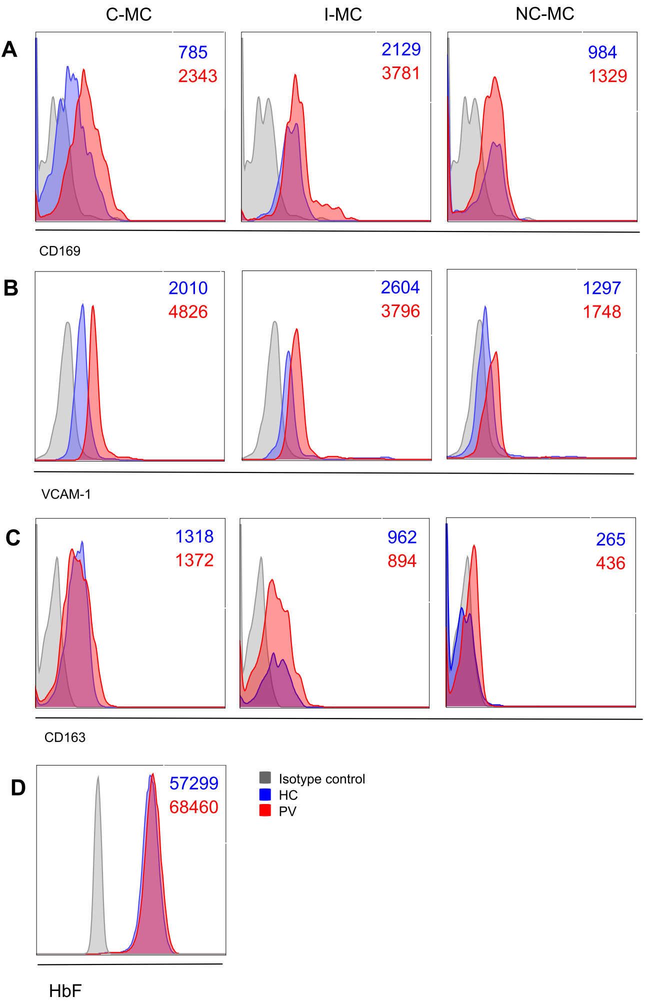
C-MC
I-MC
NC-MC
A
785
2343
2129
3781
984
1329
CD169
B
2010
4826
2604
3796
1297
1748
VCAM-1
C
1318
1372
962
894
265
436
CD163
D
57299
68460
HbF
Isotype control
HC
PV

Antibody	Clone	Function	Dilution	Company
CD14-FITC	Tuk4	Glycoprotein receptor	1:20	Life Technologies
CD16-PerCP	3G8	Type I transmembrane receptor	3:100	Life Technologies
CD169-647	7-239	Adhesion to erythroid cells	1:25	BD
CD163-BV421	GHI/31	Receptor of hemoglobin-haptoglobin complex, adhesion to erythroid cells	3:100	BD
Sirp-α-PE	SE5A5	Phagocytosis signaling	3:100	BD
VCAM1-BV605	51-10C9	Adhesion to erythroid cells	1:20	Biolegend
CD206-PE	19.2	Mannose receptor, present in regulatory cells	1:20	BD
Ferroportin- Unconjugated		Transmembrane protein that transports iron	1:100	LifeSpan Bioscience
Goat anti-Rabbit IgG (H+L) Cross-Adsorbed Secondary Antibody, Alexa Fluor™ 633	-	-	1:1000	Invitrogen
Heme-Oxigenase-1-647	23	Enzyme that breaks down heme	3:100	BD
HbF-FITC	HBF-1	Fetal hemoglobin	3:100	Life Technologies
Glycophorin-PE	CLB-ery-1	Protein found in the cell membrane of red blood cells	3:100	Life Technologies
Human Fc Block	-	-	1:20	BD
